# Tooth brushing performance in adolescents as compared to the best-practice demonstrated in group prophylaxis programs: an observational study

**DOI:** 10.1186/s12903-021-01692-z

**Published:** 2021-07-20

**Authors:** Zdenka Eidenhardt, Alexander Ritsert, Sadhvi Shankar-Subramanian, Stefanie Ebel, Jutta Margraf-Stiksrud, Renate Deinzer

**Affiliations:** 1 Department of Medical Psychology, Department of Medicine, Justus-Liebig-University Giessen, Klinikstr. 29, 35392 Giessen, Germany; 2 Department of Psychology, Philipps University of Marburg, Gutenbergstr. 18, 35032 Marburg, Germany

**Keywords:** Behavioural science, Oral health education, Community dentistry, Preventive dentistry, Oral hygiene, Dental hygiene, Tooth brushing, Oral hygiene behaviour, Observational study

## Abstract

**Background:**

Research indicates that adolescents may have difficulties to adopt the tooth brushing recommendations delivered in prophylaxis programs. However, it is not clear whether these difficulties are seen amongst the entire age range of adolescence (10–19 years) or only occur at certain developmental stages of the adolescence. The present study analyzes the tooth brushing performance of adolescents and compares it to the best-practice of tooth brushing demonstrated during prophylaxis programs.

**Methods:**

A random sample of N = 66 adolescents, comprising 10-year-olds (n = 42) and 15-year-olds (n = 24), were asked to perform oral hygiene to the best of their abilities in front of a tablet camera. Videos were analyzed for tooth brushing duration, location, and brushing movements, and the difference between the actual and expected behaviour was tested for consistency using repeated measures ANOVAs and Student’s t-tests. For the direct comparison across different age groups, already available data from 12- and 18-year-olds were reanalysed.

**Results:**

The average brushing time (mean ± SD) of the 10-year-olds and 15-year-olds was 195.8 s (74.6 s) and 196.1 s (75.8 s), respectively. Regardless of age, the adolescents distributed their brushing time unevenly across the inner, outer and occlusal surfaces. The inner surfaces in particular were neglected to a considerable extent, as no age group spent more than 15.8% of the total brushing time on them. Furthermore, all age groups showed a high proportion of horizontal movements on the inner and outer surfaces, regardless of the movements instructed for the respective surfaces.

**Conclusion:**

Even if adolescents brush to the best of their abilities, they neglect or skip one or many of the tooth surfaces. The reasons for the lack of compliance to tooth brushing instructions are discussed in light of the methods used in prophylaxis programs and the influence of parents.

## Background

Mild and moderate forms of gingival inflammation are an universal phenomenon in children and adolescents [1–3]. In Germany, the prevalence of gingivitis amongst the 12-year-olds is approximately 78% [4]. Persistent gingivitis is considered as a risk factor for the development of periodontitis. As a result, the prevention of periodontitis is dependent on the prior prevention of gingivitis [5]. Although mechanical plaque removal is considered as the first preventive method of choice for gingival or periodontal disease prevention [6, 7], it is not effective per se, unless it is performed with high-quality (i.e. regular and thorough) [8, 9]. More than 96% of 12-year-olds state that they brush their teeth at least once a day and over 80% brush twice a day [4]. High prevalence of gingivitis in this age group can therefore hardly be attributed to an irregular tooth brushing frequency, instead to a lack of thoroughness. In other words, brushing is regular, yet inefficient.

This is remarkable, as oral hygiene skills are not only acquired in the family environment, but also are a societal responsibility in Germany. According to social legislation, children are regularly instructed to practice proper oral hygiene behaviour within group and individual prophylaxis programs from the age, when they are enrolled in kindergarten until the age of 18 [10, 11]. To examine whether adolescents actually adopt the tooth brushing recommendations delivered through the prevention programs, so far two age groups have been investigated: 12-year-olds since group prophylaxis ends at that age and 18-year-olds, as the legally established individual prophylaxis measures end at that age. The participants in these studies were filmed while brushing their teeth and the brushing procedure was subsequently analyzed [12–14]. These analyses revealed that the participants neither followed the recommended brushing movements on the particular tooth surfaces nor brushed their tooth surfaces sufficiently long and completely.

Before it actually could be concluded from the named studies that adolescents do not adopt the recommended tooth brushing taught in prevention programs, it is important to analyze further age groups. During adolescence (according to the World Health Organisation 10–19 years) there are some developmental changes which might affect adolescents’ behaviour. It is thus possible, that 10-year-olds comply better with the recommendations than 12-year-olds. The former age group is possibly more willing to follow instructions given by the authorities (school dentists, teachers, etc.), whereas the 12-year-olds already begin to detach from these authorities [15]. Moreover, the skills necessary for brushing teeth are already completely developed by the age of 10 [16]. Another age group which might better comply are 15-year-olds. They tend to develop their physical self-concept based on physical abilities and appearance [17, 18]. This is of particular importance as the self-determined handling of personal hygiene plays a crucial role to feel fresh, clean and self-confident during this developmental phase [19–22]. Correspondingly, adolescents appear to brush their teeth not for health reasons, but to feel clean and fresh [23, 24]. Additionally, it has been observed that the 15–16-year olds have lower plaque levels (indicating improvements in oral hygiene) than the group of 11–12 year olds [25].

The aim of this present study was therefore to obtain a more comprehensive picture of the actual tooth brushing process of adolescents by supplementing already available data of the other age groups [13, 14] with those of 10- and 15-year-olds. In particular, it was tested whether these adolescents complied with the taught instructions (pertaining to how to brush their teeth) in the group prophylaxis programs or whether their performance would significantly deviate from these instructions.

## Methods

### Participants and recruitment

The data of the present analysis belong to a cross-sectional study investigating oral health and oral hygiene performance of children and their parents. Children of two age groups were examined together with one parent in the dental examination rooms of the Institute for Medical Psychology, Justus-Liebig University Giessen from August to December 2019. The present analysis focuses on the behavioural data of the children. The participants were recruited via their schools, social media, the University’ in-house email list and print media. Furthermore, as recruitment of 10th-graders turned out to be difficult, Giessen residents born in the year 2004 were sent some of the details of the study through post and invited to participate. Participants were given a monetary compensation of 50 Euros. After expressing interest, families were informed in detail about the study through a telephonic conversation and an appointment was made if their interest persisted and the inclusion and exclusion criteria were met. Inclusion criteria were: (1) 10- and 15-year-olds (± 12 months), (2) Only children who were enrolled in kindergarten in Germany (to ensure that they participated in German group prevention programs from kindergarten), (3) predominant use of a manual toothbrush (criterion: at least two thirds of all the brushing events), (4) very good skills in German (to answer questionnaires), (5) more than 20 natural teeth. Exclusion criteria were: (1) Cognitive or physical impairment that affects tooth brushing, (2) pregnancy/lactation, (3) fixed orthodontic appliances, (4) Removable prosthesis/dentures, (5) Oral piercings/Dental jewelry, (6) Dental prophylaxis within the last four months, (7) Consumption of antibiotics within the past three months, (8) Training of the parents in any of the dental profession. Though it was planned at the beginning to assess data of 50 parent–child couples of each age group (10- and 15-year-olds), recruitment was terminated due to two subsequent serious situations. Firstly, a cyber-attack in December 2019 led to a complete shutdown of the network and all computers of the Justus-Liebig-University. It took until March to restore the network functions and to continue the study. By mid-March, however, the COVID-19 pandemic led to a nationwide lock-down in Germany and data collection was again impossible for an unforeseeable time. As a result, the intended sample size was not reached. However, a re-calculation of the power for the intended statistical analyses on the basis of the effect sizes already determined [14], with G*Power [26] showed that the reduced sample sizes would reveal a power of 1-ß = 0.80 for most analyses. The current analysis thus refers to the data of 10-year-olds (n = 42) and 15-year-olds (n = 24), which were collected before the first shutdown in December 2019. The entire recruitment process is depicted through a flow diagram (see Appendix).

### Procedures

Participants were reminded of their appointment one day in advance and instructed to refrain from oral hygiene for at least 4 h before the examination. Prior to the arrival, the child was randomly assigned to an examiner (author ZE and research assistants DB, MS) and a dentist (author AR and research assistants PH, TS). The assigned examiner welcomed the participants and obtained an informed consent for study participation. The whole procedure consisted of five steps: Assessment of some questionnaire data, 1st clinical assessment (plaque, papillary bleeding), video observation of oral hygiene behaviour, 2nd clinical assessment (dental status, plaque) and additional questionnaires. The current analysis focuses only on the behavioural data. Participants were placed in front of a washbasin and a tablet computer with a front camera that also served as a mirror. A red transparent sheet covered the surface of the tablet computer so that the plaque staining (Miradent Mira-2-Ton®^1^) applied during the first dental examination was invisible to the participants. Three additional cameras in the room served as a back-up when the primary camera (i.e., the tablet computer) did not record the brushing performance properly. Participants were provided with a standard manual toothbrush (Elmex InterX Kurzkopf medium^2^) and toothpaste (10-year-olds: Elmex Junior^2^; 15-year-olds: Elmex Kariesschutz^2^). As children under 12 years of age should refrain from using interdental aids [27], only the 15-year-olds were additionally offered interdental aids (waxed and unwaxed dental floss: Elmex^2^, super floss: Meridol^2^), interdental brushes (Elmex^2^: size 2 and 4) and interdental sticks (TePe^3^). The participants were told that they could use any or all of the cleaning devices to the extent they wished and were left alone in the room after that. The examiner then instructed them from an adjacent room via microphone to clean their teeth to the best of their abilities (directly translated instruction: “Clean your teeth as thoroughly as possible so that they are completely clean!”). Participants gave a signal when they had finished tooth brushing and further assessments (see above) were taken immediately afterwards.

### Behavioural data: observed oral hygiene performance

The videos were analyzed according to the methods published by the group of Deinzer [13, 14] using the software Mangold INTERACT® 18 (Mangold International GmbH, Arnsdorf, Germany) to assess the following parameters: (1) *Tooth contact time* (time when the toothbrush touches the teeth, without any interruptions like spitting, rinsing etc.). (2) Tooth contact on the *occlusal, inner,* or *outer surfaces*. (3) The *sextant* of the tooth contact for the inner and outer surfaces. For outer surfaces the two antagonistic sextants were coded when children brushed with closed mandibles. (4) The *quadrant* of the tooth contact for occlusal surfaces only; (5) The brushing movements as either *horizontal, vertical, circular, Modified Bass Technique* or *no brushing movement at all.* Brushing movements were not coded at occlusal surfaces, as in general no movements other than horizontal movements were seen on these surfaces in previous studies.

Training and calibration of the examiners (authors SS, SE, AR and research assistant KB) was similar to that of previous studies [13, 14]. To code the different behavioural categories the examiners watched the video several times (for some categories also in a fixed slow motion). The categories were observed in the sequence defined above—starting with the tooth contact time and ending with the brushing movements. Calibration was provided by 10 different videos of individuals that were not involved in the present study and was considered successful after intra class correlations (ICCs) reached the calibration criterion (ICCs ≥ 0.9). Since the accuracy of the observation of tooth contact time determined all other subsequent observations, this parameter was double coded by two different persons (SS and MS) and turned out to be very accurate (ICCs > 0.998). The other categories were single-coded (surfaces: SE; sextants/quadrants: AR; movements: KB). To ensure that observations remained reliable over time during the process of analyses, randomly selected videos were observed by another person calibrated to the respective parameter (surfaces: research assistant WP; sextants/quadrants: TS; movements: WP) and blinded to the analysis of the other examiner. The intra-class coefficients of double coding of 13 videos (10 videos of the 10-year-olds and 3 videos of the 15-year-olds) were at least ICCs ≥ 0.940 for all categories. In addition, the consistency of the 3 randomly selected videos of the 15-year-olds was examined using Cohen’ s Kappa as well as visually inspected by SE and were found to be good.

Except for the dentist AR, all other examiners were completely blinded with respect to clinical or questionnaire data. In order to minimize bias due to knowledge of the clinical data, AR did the coding several months (April–May 2020) after the clinical data assessment.

### Clinical data

Current gingival health was assessed during the 1st clinical assessment using the papillary bleeding index by Saxer and Mühlemann (PBI; [28]) as modified by Rateitschak [29] on all inner (palatinal/lingual) and outer (vestibular) surfaces. Each of the surfaces was scored from 0 to 4 (0 = no bleeding, 1 = single bleeding point, 2 = several bleeding point or thin line, 3 = interdental triangle filled with blood, 4 = profuse bleeding). Dental health of the participants was assessed by the dmf-t/DMF-T. Additionally, for each tooth of the 10-year-olds it was recorded whether the tooth is deciduous, permanent, mobile, or erupting. Examiners were calibrated according to the procedures described in [30]. Briefly, they were instructed by an experienced examiner and then assessed participants not involved in the current analysis. Calibration was considered successful if more than 90% of the scorings were identical and the remaining 10% never deviated by more than 1 score in 5 subsequent patients. The examiners were blinded with respect to the oral hygiene behaviour and the questionnaire data of the children as were the participants with respect to their clinical data.

### The best practice as demonstrated by the tooth brushing song

The group prevention program in Germany starts from the kindergarten and is continued until the 6th grade (i.e., the age of 12). In many areas, including where the study took place, the programs comprises of a tooth brushing song [31] which is used to demonstrate the children the best practice of tooth brushing during the group prophylaxis sessions. This is not exclusively an educational tool. It also provides the benchmarks allowing for a detailed analysis of the degree to which the children comply outside the prophylaxis sessions to the best practice. These benchmarks have been applied for behavioural analyses in the present and former studies [14, 32]. The song is available online in a video format so that children, their parents and their teachers could see it whenever they want to. Each verse of the song pertains to one surface and begins with a few bars instructing the children to put the brush at the intended start position. Then each verse is continued by refrains, instructing the brushing movements. A refrain lasts for a duration of 7.5 s. The song begins with the occlusal surfaces. Four refrains instruct the children to brush the four occlusal quadrants by horizontal movements. It then continues with the outer surfaces, where 3 refrains instruct the children to brush whilst closing their jaws (so called tiger bite) with circular movements. Thus, here they brush two antagonistic sextants at a time. The last verse refers to the inner surfaces. At the end of the song, it is explicitly recommended to repeat the song if considered necessary by instructors, teachers or parents. A child complying with the instructions of the song brushes his or her teeth by at least 97.5 s (7.5 s per refrain, 13 refrains). It brushes its inner surfaces twice as long as the outer surfaces and 1.5 times as long as occlusal surfaces. Within a surface it brushes all sextants/quadrant for an equal duration. It employs vertical movements whilst brushing the inner surfaces and circular movements whilst brushing the outer surfaces. It also brushes the outer surfaces in the tiger bite. These are the benchmarks used in the present analyses to test whether the children comply with the tooth brushing instructions.

### Statistics

All analyses were computed using IBM SPSS Statistics version 26.0 (IBM Corporation, U.S.A). Statistical significance was set at p ≤ 0.05. All variables were tested for normal distribution by the Kolmogorof-Smirnov goodness-of-fit test and Shapiro Wilk. Invisibility for more than 5% of tooth contact time led to exclusion from further analyses. Participants who showed outlying values in tooth contact time or its percentage distribution across the surfaces (deviation of ± 3 SD from the respective group mean) were also excluded from analyses (to ensure that this definition of outliers did not bias results, all analyses were also run without such exclusion and with even more strict exclusions; see Appendix). Violation of normal distribution assumption led to additional non-parametric analyses. The following tests were run to test for compliance with the respective benchmarks:

1. Benchmark: Tooth contact time of at least 97.5 s: the number of children who brushed below the benchmark duration of 97.5 s was determined.
2. Benchmark: Distribution of the tooth contact across the surfaces by the ratio of 1:1.5:2 on the outer, occlusal and inner surfaces respectively. The tooth contact of the outer surfaces was multiplied by 2 and that of the occlusal surfaces by 1.5 and that of the inner surfaces by 1 (no change). This should have resulted in equal values of the converted variables, which was tested with a repeated measures ANOVA, corrected by Greenhouse Geisser's ε to counteract violations of the sphericity assumption.
3. Benchmark: Even distribution of tooth contact time over the 16 surfaces of the inner and antagonistic outer sextants and quadrants. Unless the rejection of the null-hypothesis of the ANOVA for the test Benchmark 2, a further ANOVA examined this Benchmark.
4. Benchmark: Predominant brushing of inner surfaces by vertical movements and of outer surfaces by circular movements. Student’s t-test were computed to test whether the expected movement exceeded that of alternative movements. Compliance with movements was assumed when both t-test revealed a significant result.
5. Benchmark: Brushing outer surfaces with mandibles closed (tiger bite). Student’s t-test for dependent measures to see whether tooth contact with mandibles closed exceeds that with mandibles not closed on the outer surfaces.

As ŋ^2^ overestimates the population effect size [33], ANOVAs are additionally reported together with Effect size f. According to 34 [34] Effect sizes of ƒ ≥|.10| |.25| |.40| are considered small, medium and large, respectively. Student’s t-tests are reported along with Cohen’s d (difference of means divided by pooled standard deviation) as a measure of the effect size. According to 34 [34] effect sizes of d ≥|.2| |.5| |.8| are considered small, medium and large, respectively.

To further explore the brushing performance and compare it with the other groups [13, 14] the following variables were additionally analysed and depicted descriptively: (1) Percentage of tooth contact time spent brushing the inner, outer and occlusal surfaces. (2) tooth contact on occlusal surfaces of the quadrants. (3) Tooth contact on inner and outer sextants using the Quality Index of tooth brushing regarding brushing time in sextants (QIT-S, 13) on the inner and outer surfaces. The QIT-S scores vary from 0 to 9. The highest score (9) is given, when tooth contact within the respective surface equals at least 7.5 s for all sextants. Scores 8, 7, and 6 indicate that all 6 sextants were brushed for at least 5 s, 3.5 s, or more than 1 s, respectively. Scores below 6 indicate total neglect (no more than 1 s tooth contact) of 1(5), 2(4), 3(3), 4(2) 5(1), or 6(0) sextants, respectively.

To further complement the analyses, data from previous studies with 12- and 18-year-olds [13, 14, 32] were reanalyzed to allow for direct comparison across different age groups based on some descriptive characteristics. Only those participants who had received the same brushing instruction (brushing to the best of one´s abilities) were included into this comparison.

The study conforms with STROBE (Strengthening the Reporting of Observational Studies in Epidemiology) guidelines.

## Results

### Characteristics of the sample

In total, 42 10-year-olds and 24 15-year-olds provided data for this analysis. Table 1 shows the characteristics of the sample. 50% (n = 21) of the 10-year-olds and 33.3% (n = 8) of the 15-year-olds had neither filled nor decayed teeth. Mean dmf-t/DMF-T for the 10- and 15-year-olds was *M* = 1.5 (± 2.5) and *M* = 2.3 (± 2.6), respectively. 54.8% (n = 23) of the 10-year-olds and 79.2% (n = 19) of the 15-year-olds had at least one of their teeth sealed. None of the 10- and 15-year-olds was found to be free of papillary bleeding.

**Table 1:** Characteristics of the sample

	10-year-olds (n = 42)	15-year-olds (n = 24)
	*M* (± *SD*) [min, max] n/n	*M* (± *SD*) [min, max] n/n
Age	10.1 (0.5) [9, 11]	15.2 (0.4) [15, 16]
Sex (female/male/non-binary)	21/21/–	6/17/1
Educational status of parents		
UED^a^	36	15
No UED	6	8
Unknown	–	1
Dental status		
Permanent teeth	16 (4.7) [10, 25]	27.9 (0.5) [26, 29]
Deciduous teeth^b^ (0/1–5/6–10/11–13 teeth)	7/9/11/15	22/2/–/–
Erupting teeth (0/1–3/4–5 teeth)	27/13/2	N/A
Mobile teeth (0/1–3/4–5 teeth)	36/6/–	N/A
dmf-t/DMF-T (0/1–2/3–6/7–12)	21/14/5/2	8/8/5/3
Decayed teeth (0/1–2/3–4/5–8 teeth)	28/11/3/–	19/4/1/–
Filled teeth (0/1–2/3–4/5–8 teeth)	29/8/3/2	9/8/4/3
Papillary bleeding^c^		
Mean overall score		
Total	0.8 (0.4) [0.1, 1.8]	0.9 (0.5) [0.1, 1.9]
Inner surfaces	0.9 (0.5) [0.2, 2.0]	1.0 (0.5) [0.2, 1.9]
Outer surfaces	0.7 (0.5) [0.0, 1.9]	0.7 (0.6) [0.0, 2.0]
Mean percentage of sides bleeding		
Total	50.5 (21.5) [6.3, 94.0]	49.9 (23.4) [8.9, 94.6]
Inner surfaces	54.8 (22.4) [12.5, 92.0]	58.6 (23.0) [14.3, 100]
Outer surfaces	46.2 (25.5) [0.0, 96.0]	41.1 (27.9) [3.6, 89.3]

^a^UED: at least one parent with university entrance diploma

^b^N = 2 15-year-olds had one and two deciduous teeth, respectively

^c^Papillary Bleeding Index (PBI) expressed as mean overall score (*M*) and mean percentage of sites bleeding (%)

### General description of tooth brushing behaviour

**Fig. 1:**
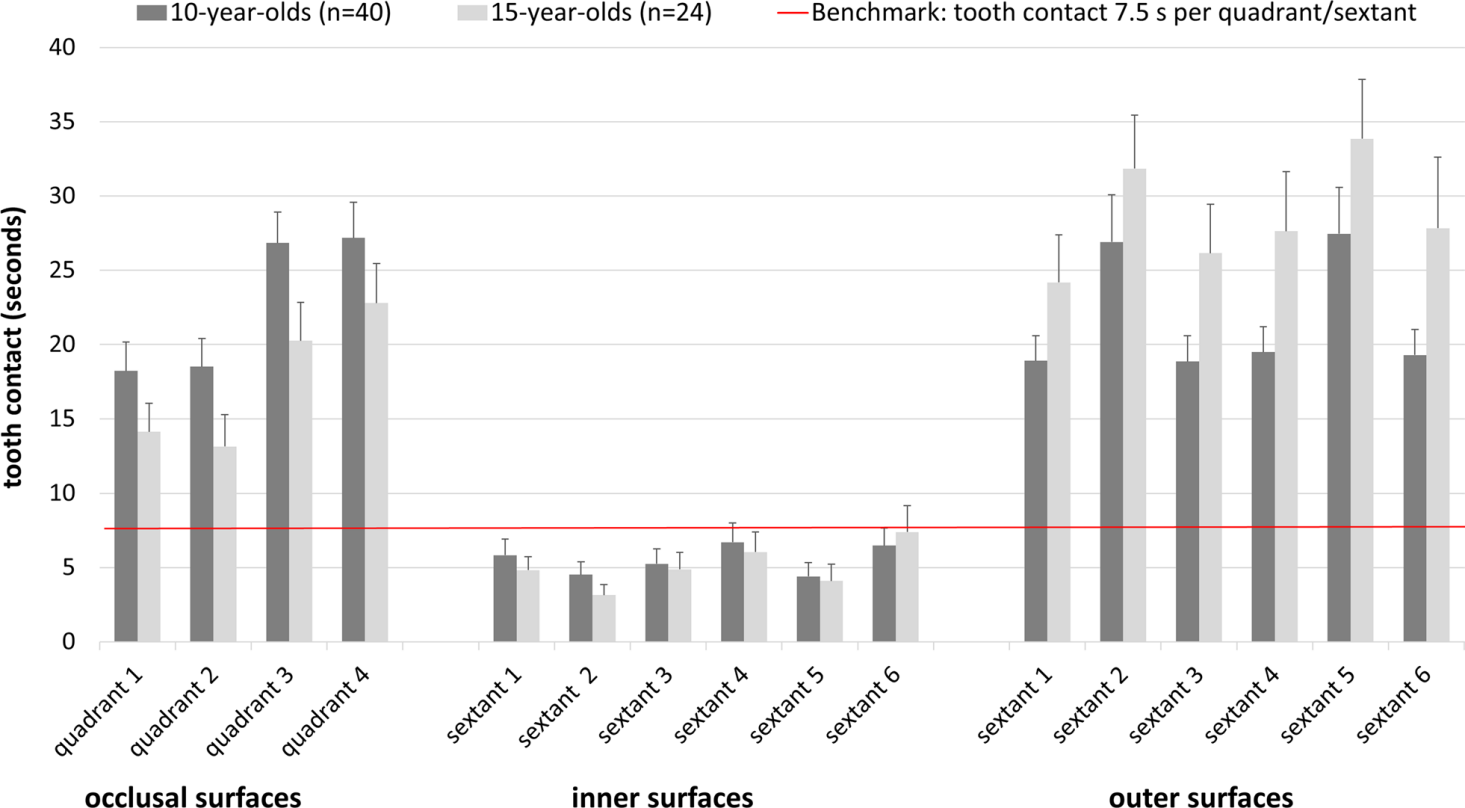
Means and standard error of the means of the duration of tooth contact on occlusal surfaces (quadrant 1–4) and on inner surfaces (sextant 1–6) and outer surfaces (sextant 1–6). Tooth contact while brushing with mandibles closed is attributed to both antagonistic sextants

**Fig. 2:**
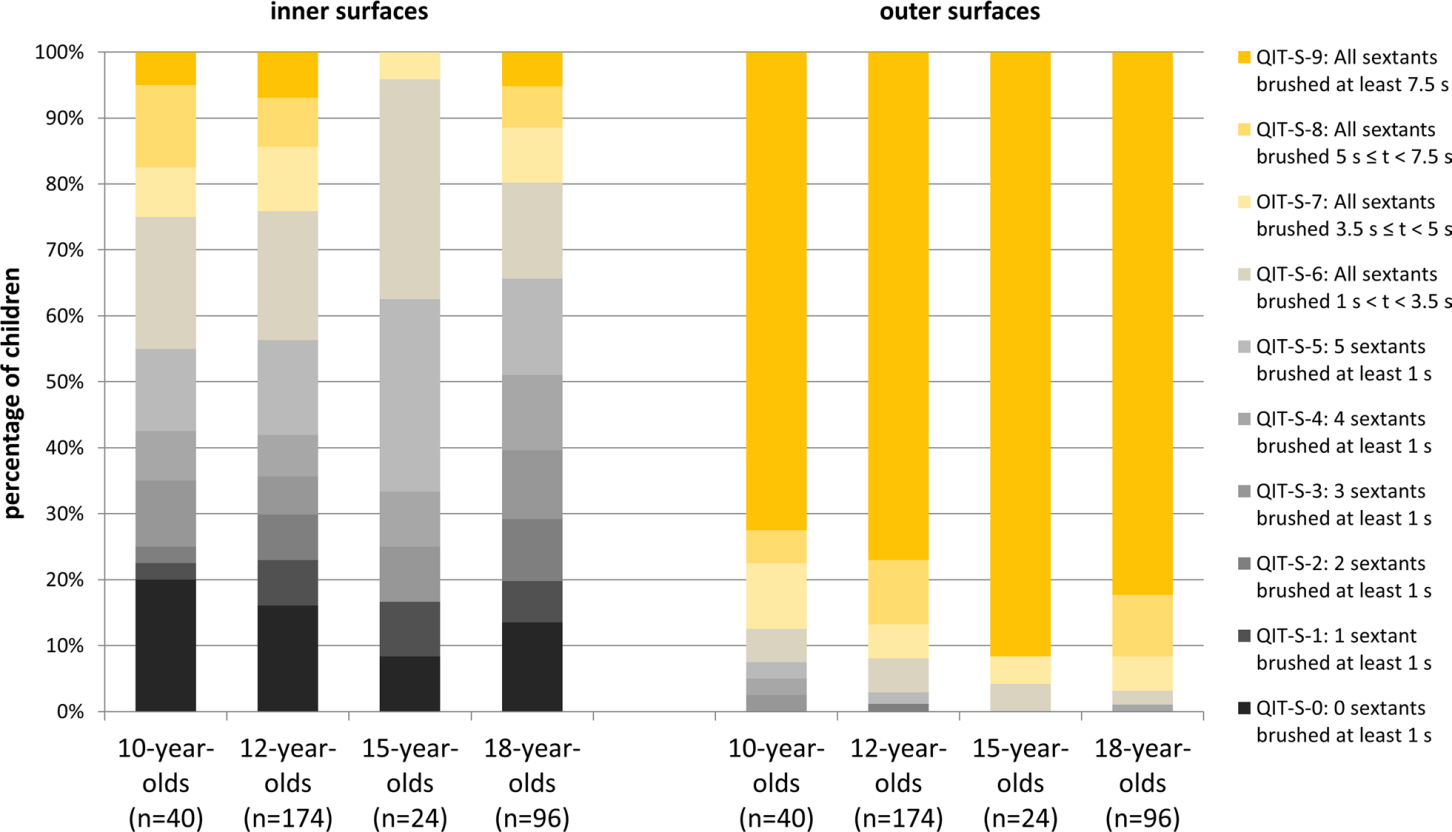
QIT-S scores indicating tooth contact on inner and outer surfaces of sextants for 10- 12-, 15- and 18-year-olds respectively (Figure includes data from the studies of 13, 14)

N = 10 of the 15-year-olds used interdental hygiene aids (these were not offered to the 10-year-olds). None of the participants had to be excluded because of extended periods of non-visibility. Only two participants were invisible for a short period of time (< 0.5% of tooth contact time). N = 2 10-year-olds had to be excluded from further analyses as their tooth contact time and tooth contact on the outer surfaces exceeded the respective group means by 3 SDs. Table 2 shows the behavioural parameters regarding tooth contact time and brushing movements. Figure 1 displays the distribution of tooth contact across the quadrants and sextants on the inner and outer surfaces. Analyses regarding occlusal surfaces showed that 80% (n = 32) 10-year-olds and 54.2% (n = 13) 15-year olds brushed all quadrants for at least 7.5 s. Figure 2 shows the QIT-S-scores for inner and outer surfaces. 5% of the 10-year-olds (n = 2) and none of the 15-year-olds brushed all of the inner sextants for at least 7.5 s, whereas 20% (n = 8) of the 10-year-olds and 8.3% (n = 2) of the 15-year olds completely neglected them. Outer sextants were brushed for at least 7.5 s by 72.5% (n = 29) 10-year-olds and by 91.7% (n = 22) 15-year-olds (see Fig. 2). None of the adolescents brushed either all of the sextants (inner and outer) or all of the quadrants for 7.5 s. N = 8 10-year-olds (but none of the 15-year-olds) brushed all these surfaces for at least 5 s.

**Table 2:** Tooth brushing performance of 10- and 15-year-olds

	10-year-olds (n = 40)	15-year-olds (n = 24)
	*M* (*SD*); *Mdn* (Q1, Q3)*	*M* (*SD*); *Mdn* (Q1, Q3)*
Tooth contact time (s)	195.8 (74.6)	196.1 (75.8)
Inner surfaces	33.2 (33.7); 19.3 (5.7, 62.2)	30.4 (26.7)
Outer surfaces	71.9 (36.2)	95.3 (54.9); 78.2 (57.7, 125.3)
Occlusal surfaces	90.7 (42.7)	70.3 (36.0)
% of tooth contact time		
Inner surfaces	15.3 (13.8); 13.2 (2.8, 25.1)	15.8 (11.5)
Outer surfaces	36.9 (13.1)	47.4 (14.7)
Occlusal surfaces	47.8 (16.6)	36.8 (13.3)
% of movements on inner surfaces^a,b^		
Circular^c^	1.0 (5.6); 0.0 (0.0, 0.0)	0.7 (3.3); 0.0 (0.0, 0.0)
Horizontal	54.6 (39.7); 62.4 (7.2, 93.8)	68.8 (36.2); 85.9 (32.0, 98.2)
Vertical	44.3 (40.2); 33.0 (6.2, 92.8)	30.5 (35.2); 14.1 (1.8, 67.6)
No movements^d^	0.2 (0.5); 0.0 (0.0, 0.0)	N/A
% of movements on outer surfaces^b^		
Circular	39.9 (35.9); 27.0 (0.0, 78.9)	44.0 (36.6); 46.4 (0.3, 75.8)
Horizontal	53.7 (37.9); 55.0 (13.9, 95.6)	51.1 (39.2); 42.7 (17.5, 98.4)
Vertical^e^	5.2 (17.2); 0.0 (0.0, 0.8)	4.5 (9.7); 0.0 (0.0, 3.7)
No movements^d^	1.2 (2.1); 0.0 (0.0, 1.3)	0.4 (0.7) 0.0 (0.0, 0.8)

*Median and 1st and 3rd quartile are additionally reported if the normal distribution is violated

^a^Not including those 10-year-olds (n = 8) and 15-year-olds (n = 2) who did not brush their inner surfaces at all

^b^None of the adolescents showed Modified Bass Technique

^c^In both age groups, only one participant showed circular movements at inner surfaces

^d^Only n = 4 10-year-olds showed for short periods of time (min = 0.9%, max = 2.0%) tooth contact without any movements at inner surfaces. At outer surfaces tooth contact without any movements was observed within n = 20 10-year-olds (min = 0.2%, max = 9.2%) as well as n = 8 15-year-olds (min = 0.6%, max = 2.2%)

^e^Only n = 11 10-year-olds (min:0.8%, max:100%) and n = 9 15-year-olds (min:1.1%, max:37.7%) showed vertical movements on outer surfaces at all

### Adolescents’ compliance with tooth brushing instructions

Benchmark 1 (≥ 97.5 s tooth contact time) was exceeded by almost all children; only one 15-year-old was below that value (83.9 s). Benchmark 2 (distribution of brushing time by 1:1.5:2 to outer, occlusal and inner surfaces, respectively) was neither met by the 10-year-olds (F(2/78) = 52.20, p < 0.001, ε = 0.953, ŋ^2^ = 0.572, ƒ = 1.16) nor by the 15-year olds (F(2/46) = 31.17, p < 0.001, ε = 0.674, ŋ^2^ = 0.575, ƒ = 1.16). This made the examination of Benchmark 3 (distribution across all 16 areas) redundant. Benchmark 4 (circular movements at outer and vertical movements at inner surfaces) was also neither met by the 10- nor by the 15-year-olds (see also Table 2). Instead, horizontal movements predominated in both the age groups on the inner (10-year-olds: d = −0.26; 15-year-olds: d = −1.16) and outer surfaces (10-year-olds: d = −0.37; 15-year-olds: d = −0.19). Benchmark 5 (predominant brushing of outer surfaces with mandibles closed) was met by both the 10-year-olds (t(39) = 7.876, p < 0.001, d = 2.49) and 15-year-olds (t(23) = 6.622, p =  < 0.001, d = 2.70). Due to violations of the normal distribution assumption, additional non-parametric statistics were calculated. Since these analyses revealed comparative results (all p < 0.001), these tests are not presented in detail here.

### Comparison of the tooth brushing performance of four age groups (10-, 12-, 15- and 18-year-olds)

**Fig. 3:**
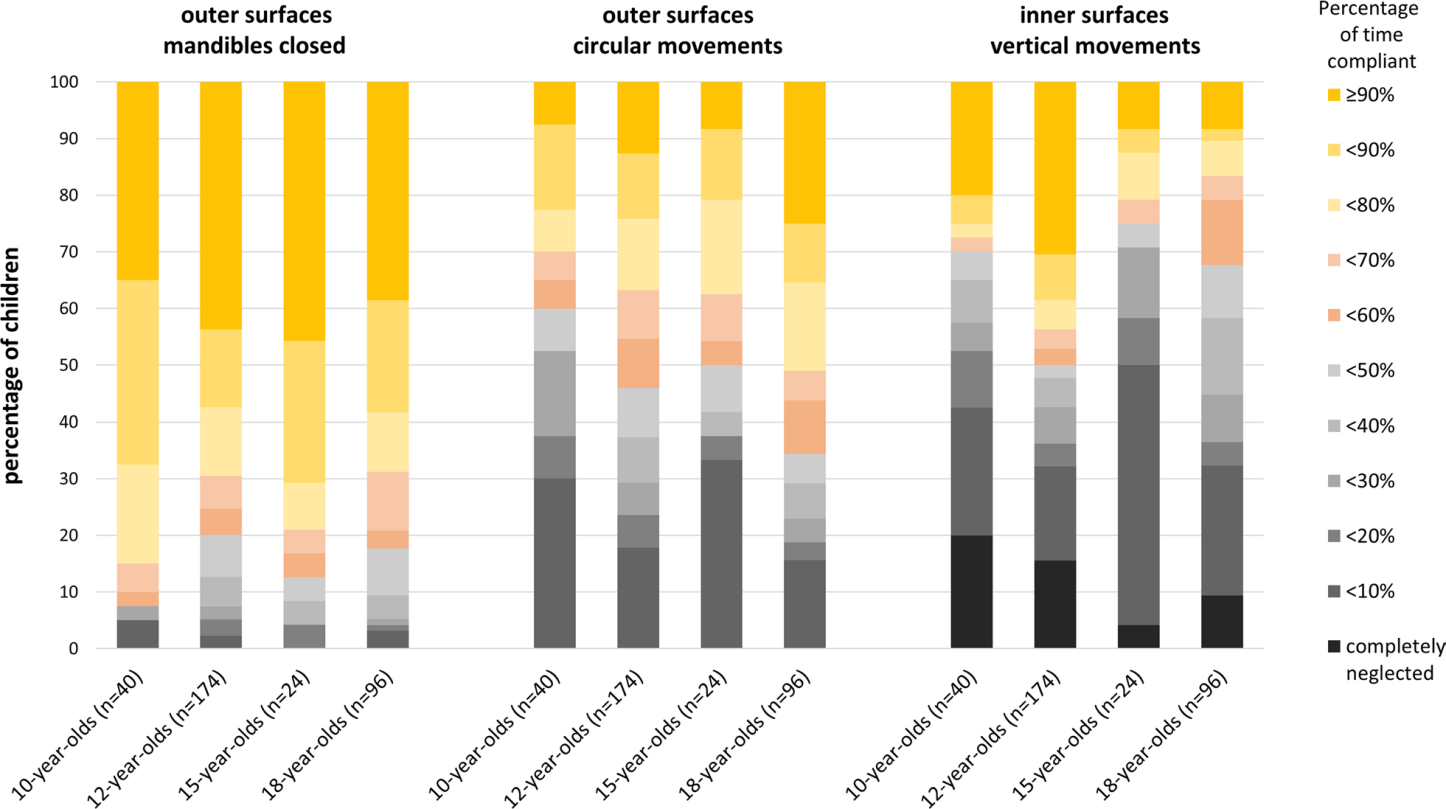
Distribution of the percentage of tooth contact time by which adolescents complied to the respective brushing recommendations given in the brushing song (Figure includes data from the studies of 13, 14)

Tooth contact time and the distribution of brushing movements and tooth contact on inner, outer and occlusal surfaces form the previous studies of the 12-year-olds [14, 32] and 18-year-olds [13] are presented in the Appendix. Figure 2 shows the distribution of QIT-S-scores across all four age groups. Further, Fig. 3 shows the respective extent of compliance with the brushing recommendations pertaining to brushing movements and brushing outer surfaces in tiger bite for all four age groups. A compliance ≥ 90% of tooth contact time for both vertical movements on inner surfaces and circular movements on the outer surfaces was shown by 2.5% (n = 1) 10-year-old, 7.5% (n = 13) 12-year-olds, none 15-year-old and 3.1% (n = 3) 18-year-olds.

## Discussion

The present study examined whether adolescents comply to the tooth brushing instructions given in the group prophylaxis programs. Therefore, two age groups were observed who brushed their teeth to the best of their abilities. The degree of compliance was determined by categorizing the observed brushing performance and comparing it with the performance that could be expected from the delivered instructions in the programs. The tooth brushing song (a didactic tool of group prophylaxis) defined the standard for the expected performance and formed the core of the compliance analyses. Due to particular developmental changes during adolescence, the 10- and 15-year-olds were expected to show good compliance with these benchmarks. Firstly, the results were evaluated with regards to this assumption by comparing the exhibited brushing performance of the two age groups recruited in this study. Secondly, the results were compared with the already investigated age groups: 12-year-olds [14] and 18-year-olds [13].

The average tooth contact time in both the age groups was more than three minutes. With a brushing time more than twice of the defined duration in the benchmark, the adolescents could be considered to comply with the first benchmark. Nevertheless, they brushed irrespective of their age, the inner surfaces shorter than the other surfaces. In contrast, they were expected to brush them twice as long as the outer surfaces, at least when they brush outer surfaces in the tiger bite (which most of them did for most of the time, so they are also compliant with the fifth benchmark). However, children not only brushed their inner surfaces for less time than the other surfaces. In addition, none of the 15-year-olds and only two of the 10-year-olds brushed the inner surfaces of all the sextants sufficiently long (at least 7.5 s) and 21.4% of the 10-year-olds and 8.3% of the 15-year-olds completely neglected them. On the contrary, 91.7% of the 15-year-olds and 72.9% of the 10-year-olds sufficiently brushed all the sextants of the outer surfaces by at least 7.5 s (cf. Fig. 2). Here, a considerable difference between the two age groups became obvious. Total neglect of inner surfaces and partial neglect of the outer surfaces is seen more often in 10- than in 15-year-olds. Another difference is observed with reference to the occlusal surfaces. While 10-year-olds spent almost half of the time brushing their occlusal surfaces, the 15-year-olds focused on the outer surfaces (cf. Table 2). The attention that 15-year-olds paid to the visible outer surfaces is in agreement with the presumption that grooming and physical appearance might motivate and guide tooth brushing at this age [35]. Despite the observation that the 15-year-olds brushed their occlusal surfaces for less time than the 10-year-olds, both the age groups showed a similar brushing pattern here: The occlusal surfaces of the maxilla were brushed for less time than those of the mandible (cf. Fig. 1). A further similarity could be seen in the brushing time of sextants 2 and 5. While both age groups brushed the outer surfaces of these two sextants for a longer duration, they also brushed their inner surfaces for less than the respective surfaces of the other sextants. Whilst the former observation is probably most likely due to the good visibility of the anterior teeth, the latter could be due to the horizontal movements that were mainly performed on the inner surfaces (these are hardly feasible in sextants 2 and 5). At this point, we conclude that the 10- and 15-year-olds do not meet the second and the third benchmarks (the even distribution of brushing time across the respective surfaces). Regarding the fourth benchmark (the surface-specific movements), only one 10-year-old (and none of the 15-year-olds) showed a good compliance (≥ 90%) with both circular movements on the outer surfaces and vertical movements on the inner surfaces. Even though the circular movements on the inner surfaces were almost never seen and vertical movements on the outer surfaces were also rare, horizontal movements predominated on both the surfaces. Remarkably, the 15-year-olds showed clearly fewer vertical movements on the inner surfaces than the 10-year-olds, who seemed to better comply to this particular instruction than the older adolescents did (cf. Table 2 and Fig. 3). To summarize, the 10- and 15-year-olds, who brushed their teeth to the best of their abilities, took enough time to accomplish this task. While they spent more than twice of the instructed brushing duration in the group prophylaxis, not a single adolescent managed to brush all of the tooth surfaces for a sufficient time. In addition, only one of the adolescents brushed most of both, the inner and outer surfaces with the recommended movements.

The overall objective of the present study was to supplement the existing knowledge regarding tooth brushing performance in adolescents and thus to obtain a comprehensive picture of whether, when and to what respect adolescents adopt the instructions of group prophylaxis. For this purpose, these results are further compared with the results of the 12- and 18-year-olds, that were examined with comparable methodological standards [13, 14]. Regarding average brushing time, there is an agreement of all age groups; they show average brushing times of more than 3 min, at least when asked to brush to the best of their abilities. Irrespective of this instruction, there is a considerable neglect of the inner surfaces in all age groups. None of the age groups brushed their inner surfaces by more than 15.8% of the total brushing time and only a small minority (< 7%) of each age group managed to brush all of the inner sextants by at least 7.5 s. The overall comparison shows that 12-year-olds are starting to pay more attention to their outer surfaces than to the occlusal surfaces (cf. Table in the Appendix). A similar pattern is seen amongst the 15- and 18-year-olds. This might indicate that from the age of 12 onwards, awareness of personal appearance and grooming develops and thus changes the focus of tooth brushing. Despite the observation that from a certain age, tooth brushing seems to serve a well-groomed appearance, at no point in adolescence does an awareness develop of how long a particular surface needs to be brushed. Just as striking as the neglect of the inner surfaces, is the preference for horizontal movements in all age groups. Although there is a tendency to use the recommended movements for the respective surfaces, a large proportion of horizontal movements on the inner and outer surfaces could be observed in all age groups. On the one hand, the 12-year-olds seem to adopt the required movements better than the other age groups. On the other hand, even in this age group only 7.5% performed the recommended movements for most of the time on both the inner and outer surfaces. However, a trend could be seen in the data. With increasing age, the readiness to brush the outer surfaces with circular movements seems to increase. Given that tooth brushing is considered a routine behaviour [36], this observation could indicate that circular movements are incorporated into the so-called script of tooth brushing. Concerning vertical movements, the trend seems to be opposite: Even if the compliance for applying vertical movements on the inner surfaces increases from 10 to 12 years, it then decreases continuously (cf. Fig. 3). Apparently, the movements that are learned once are discarded again. This could perhaps be due to convenience, as the horizontal movements are performed with less effort than the more complex vertical movements [37].

In conclusion, the efforts of group prophylaxis seem to have developed an awareness of the significance of tooth brushing. However, even when participants are brushing at the best of their abilities, this awareness is mainly expressed through an increase in the duration of the overall tooth brushing. Regardless of age, there is no awareness of the completeness of brushing or the need for specific brushing movements on the respective surfaces. The most outstanding aspect is the neglect of the inner surfaces, which, if at all, are brushed predominantly with horizontal movements.

The question arises as to why there is a lack of compliance. Even though only users of manual toothbrushes were considered in this paper, it has to be emphasized that comparable results were found for users of powered toothbrushes [38, 39]. As favourable outcomes are described for powered toothbrush users in terms of tooth brushing consistency when supported by a smartphone application [40, 41], the question arises, if methods (other than a tooth brushing song) should facilitate tooth brushing instructions. Due to their ease of use and availability at any time, such applications could also be of particular value to manual toothbrush users, as repetition and reinforcement are important elements for the sustainability of oral health education programs [42]. Regardless of the media supporting the instruction, the delivery mode also matters, as experiential learning [43] and individual oral health counselling [44] have been found to show improvements in tooth brushing behaviour. Since some participants showed the instructed brushing performance irrespective of their age, the problems of basic feasibility of the taught tooth brushing and age of the participants are ruled out. Given that the brushing duration exceeded the recommended duration, a lack of willingness to make an effort for proper tooth brushing also seems unlikely. In fact, the lack of compliance could neither be due to the subjects nor be due to the design of the prophylaxis programs. Regardless of the institutionalized oral health education, children's oral health behaviour essentially develops during the initial stages of development mainly in the family environment [45, 46]. If the acquired tooth brushing habits at home contradict the measures of the prophylaxis programs, learning the contents taught in the programs is impeded [47, 48]. The varying compliance to vertical movements on inner surfaces across the four age groups might be an indication of a relapse into old (or simpler) habits.

Besides the high degree of standardization and the elaborated video analyses, there are also some limitations of the study that are to be mentioned. Self-selection of study participants might have induced a selection bias. Furthermore, the study was conducted particularly in one specific area alone. However, the clinical data of this group appear to be well representative of the German samples [4, 49]. In addition, the behavioural data within the current sample match remarkably well with the data of earlier observational studies [13, 14, 38]. Therefore, results could be valid for other areas too. Another limitation that also refers to the subject population is the exclusion of study participants with a dental prophylaxis in the last 4 months, which might have skewed the population towards less caring households. Spatial ability to reach the lingual surfaces could be considered a further limitation that might affect the group of 10-year-olds in particular. However, a toothbrush with a small head was used in this study. Thus, this does not readily explain the neglect of these surfaces. Furthermore, though this study allows for the comparison of different age cohorts, one should keep in mind that firm conclusions regarding the development of tooth-brushing competences require longitudinal observations. The current analyses thus only give a first hint of what might characterize this development.

## Conclusion

In summary, the tooth brushing instructions delivered in the group- and individual prophylaxis are not adopted to a large extent. The most serious problem here is that adolescents do not adopt the concept of complete tooth brushing, i.e., brushing all sections for a sufficient time. Even if they brush to the best of their abilities, they skip and neglect some of the tooth surfaces. One reason for the lack of compliance could be due to the way the instructions were delivered in the prophylaxis programs. In addition, however, it should also be examined whether incorrect instructions by parents has already directed early childhood tooth brushing towards a wrong direction. Since within the framework of this study, in addition to the 10- and 15-year-olds, their parents were also examined, it seems worthwhile to take a closer look at the respective parents' tooth brushing behaviour [50].

## Figures and Tables

**Fig. 4:**
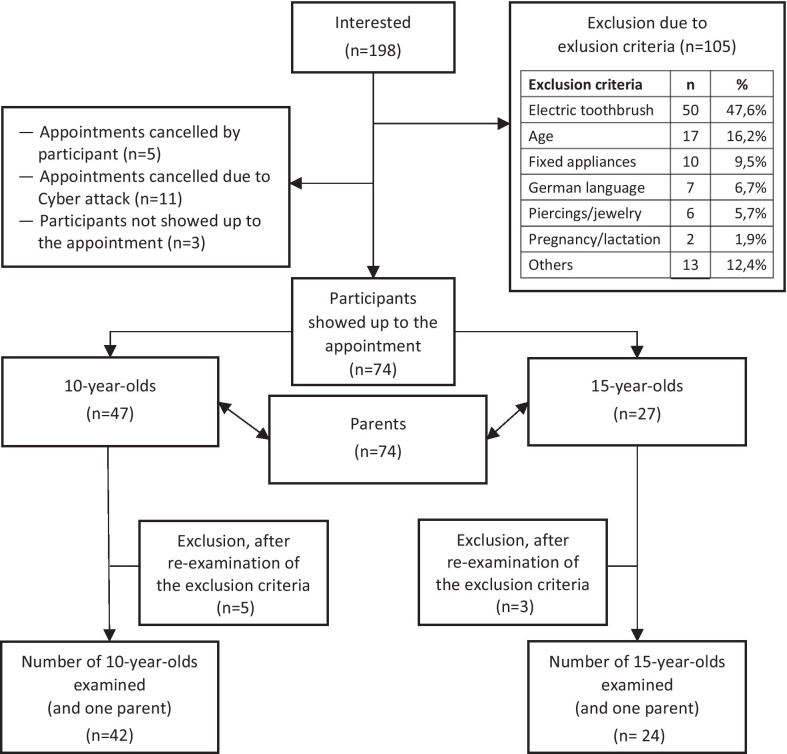
Flowchart of the recruitment

## Data Availability

The datasets used and/or analyzed during the current study are available from the corresponding author on reasonable request. However, for privacy reasons, no individual data allowing identification of participants (e.g., videos) can be provided.

## Appendix

See Tables

**Table 3:** Tooth brushing performance of 10- and 15-year olds (including all participants, N = 66)

	10-year-olds (n = 42)	15-year-olds (n = 24)
	*M* (*SD*); *Mdn* (Q1, Q3)*	*M* (*SD*); *Mdn* (Q1, Q3)*
Tooth contact time (seconds)	201.1 (87.5)	196.1 (75.8)
Inner surfaces	32.4 (33.3); 19.3 (4.6, 60.9)	30.4 (26.7)
Outer surfaces	78.8 (52.1)	95.4 (54.9); 78.2 (57.7, 125.3)
Occlusal surfaces	89.9 (44.8)	70.3 (36.0)
% of tooth contact time		
Inner surfaces	14.8 (13.8); 12.5 (2.4, 24.0)	15.8 (11.5)
Outer surfaces	39.0 (16.5)	47.4 (14.7)
Occlusal surfaces	46.2 (18.0)	36.8 (13.4)
% of movements on inner surfaces^a, B^		
Circular^c^	1.0 (5.5); 0.0 (0.0, 0.0)	0.7 (3.3); 0.0 (0.0, 0.0)
Horizontal	53.5 (39.5); 60.1 (8.5, 93.3)	68.8 (36.2); 85.9 (32.0, 98.2)
Vertical	45.4 (40.1); 35.3 (6.6, 91.5)	30.5 (35.2); 14.1 (1.8, 67.6)
No movements^d^	0.2 (0.5); 0.0 (0.0, 0.0)	N/A
% of movements on outer surfaces^b^		
Circular	38.0 (36.0); 25.7 (0.0, 78.5)	44.0 (36.6); 46.4 (0.3, 75.8)
Horizontal	55.8 (38.2); 57.3 (15.8, 97.6)	51.1 (39.2); 42.7 (17.5, 98.4)
Vertical	5.0 (16.8); 0.0 (0.0, 0.8)	4.5 (9.7); 0.0 (0.0, 3.7)
No movements	1.2 (2.1); 0.0 (0.0, 1.3)	0.4 (0.7) 0.0 (0.0, 0.8)

*Median and 1st and 3rd quartile are additionally reported if the normal distribution is violated

^a^Not including those 10-year-olds (n = 9) and 15-year-olds (n = 2) who did not brush their inner surfaces at all

^b^None of the adolescents showed Modified Bass Technique

3,

**Table 4:** Detection of outliers* in behavioural parameters of 10-year-olds (n = 8) and 15-year olds (n = 4)

Participant (no)	Total	Tooth contact time (s)			% of tooth contact time			% of movements on inner surfaces				% of movements on outer surfaces				
		Inner surfaces	Outer surfaces	Occlusal surfaces	Inner surfaces	Outer surfaces	Occlusal surfaces	Circular	Horizontal	Vertical	No movem.	Circular	Horizontal	Vertical	No movem.	% tiger bite on outer surfaces
10-year-olds																
16008	233.32	80.08	62.84	90.40	34.32	26.93	38.75	0.00	83.32	14.74	**1.95**	85.68	4.33	0.83	**9.17**	92.87
16024	378.24	65.16	131.36	181.72	17.23	34.73	48.04	0.00	29.96	68.08	**1.96**	59.07	38.48	0.00	2.50	95.55
16040	319.32	48.12	103.40	167.80	15.07	32.38	52.55	0.00	97.09	2.00	0.91	23.87	68.43	0.00	**7.70**	71.49
16046	**498.80**	34.32	**317.44**	147.04	6.88	63.64	29.48	0.00	18.18	81.82	0.00	2.22	97.56	0.00	0.23	86.50
16049	159.12	19.64	11.16	128.32	12.34	7.01	80.64	0.00	92.46	7.54	0.00	0.00	100.00	0.00	0.00	**0.00**
16068	231.72	86.12	66.64	78.96	37.17	28.76	34.08	0.00	0.00	100.00	0.00	0.00	0.00	**100.00**	0.00	**1.08**
16085	115.92	0.00	115.32	0.60	0.00	**99.48**	0.52	–	–	–	–	0.00	100	0.00	0.00	77.70
16086	252.16	65.00	74.84	112.32	25.78	29.68	44.54	**31.51**	57.72	57.72	0.00	21.75	78.25	0.00	0.00	92.20
15-year-olds																
16052	119.32	1.04	40.40	77.88	0.87	33.86	65.27	0.00	100.00	0.00	0.00	0.00	100.00	0.00	0.00	**12.08**
16053	255.32	97.60	87.00	70.72	38.23	34.07	27.70	**15.49**	13.28	71.23	0.00	85.52	12.28	0.00	2.21	95.40
16063	134.08	15.00	57.20	61.88	11.19	42.66	46.15	0.00	0.00	100.00	0.00	61.05	1.26	**37.69**	0.00	99.58
16088	291.52	7.60	**265.60**	18.32	2.61	91.11	6.28	0.00	100.00	0.00	0.00	41.97	46.14	11.27	0.62	74.82

*Outliers (deviation of  ± 3 *SD* from the mean) are highlighted in bold

4,

**Table 5:** Tooth brushing performance of 10- and 15-year olds (N = 54) with outlier correction in all behavioural parameters

	10-year-olds (n = 34)	15-year-olds (n = 20)
	*M* (*SD*); *Mdn* (Q1, Q3)*	*M* (*SD*); *Mdn* (Q1, Q3)*
Tooth contact time (s)	184.1 (68.9)	195.3 (75.0)
Inner surfaces	28.4 (33.0); 15.8 (3.0, 41.1)	30.4 (23.2)
Outer surfaces	71.3 (36.0)	91.9 (43.3); 78.2 (59.2, 125.3)
Occlusal surfaces	84.4 (40.3)	72.0 (37.5)
% of tooth contact time		
Inner surfaces	13.9 (14.0); 11.8 (1.5, 17.9)	16.3 (10.5)
Outer surfaces	38.7 (12.9)	46.8 (11.9)
Occlusal surfaces	47.4 (16.9)	36.9 (10.6)
% of movements on inner surfaces^a, b^		
Circular	N.A	N.A
Horizontal	53.3 (40.5); 62.4 (5.9, 94.0)	72.3 (32.1); 85.9 (46.9, 96.5)
Vertical	46.7 (40.6); 37.6 (5.0, 94.1)	27.7 (32.1); 14.1 (3.5, 53.1)
No movements	0.0 (0.2); 0.0 (0.0, 0.0)	N.A
% of movements on outer surfaces^b^		
Circular	41.3 (36.5); 34.3 (0.0, 79.8)	43.4 (37.6); 45.7 (0.3, 75.8)
Horizontal	54.6 (37.9); 54.9 (15.8, 96.5)	53.4 (39.0); 42.7 (20.0, 98.4)
Vertical	3.2 (8.3); 0.0 (0.0, 1.0)	2.9 (7.0); 0.0 (0.0, 1.9)
No movements	0.8 (1.3); 0.0 (0.0, 1.3)	0.3 (0.6); 0.0 (0.0, 0.8)

*Median and 1st and 3rd quartile are additionally reported if the normal distribution is violated

^a^Not including those 10-year-olds (n = 8) and 15-year-olds (n = 2) who did not brush their inner surfaces at all

^b^None of the adolescents showed Modified Bass Technique

5,

**Table 6:** Tooth brushing performance of 12- and 18-year olds

	12-year-olds (n = 174)	18-year-olds (n = 96)
	*M* (*SD*); *Mdn* (Q1, Q3)*	
Tooth contact time (seconds)	199.8 (80.5)	206.7 (84.0)
Inner surfaces	32.3 (30.8); 27.6 (6.0, 49.5)	30.6 (30.9); 21.3 (5.0, 47.6)
Outer surfaces	85.5 (46.0)	91.3 (40.7)
Occlusal surfaces	82.1 (42.5)	84.8 (46.0)
% of tooth contact time		
Inner surfaces	15.2 (12.2); 14.2 (3.7, 23.7)	13.3 (10.3); 12.9 (3.7, 21.6)
Outer surfaces	43.2 (15.1)	46.1 (16.5)
Occlusal surfaces	41.6 (15.3)	40.6 (15.3)
% of movements on inner surfaces^a^		
Circular^b^	1.0 (6.9); 0.0 (0.0, 0.0)	6.9 (16.3); 0.0 (0.0, 4.9)
Horizontal	37.2 (38.8); 20.0 (0.0, 76.0)	52.2 (30.5); 57.3 (27.1, 76.1)
Vertical	58.9 (39.0); 74.0 (20.6, 97.1)	40.7 (30.7); 37.9 (16.3, 59.5)
No movements^c^	2.8 (9.6); 0.0 (0.0, 1.8)	N/A
% of movements on outer surfaces		
Circular	50.9 (32.4); 56.0 (23.7, 79.6)	59.5 (34.0); 70.4 (33.7, 90.4)
Horizontal	38.8 (30.4); 33.6 (12.5, 60.5)	35.4 (33.2); 26.1 (5.0, 62.3)
Vertical^d^	8.3 (14.7); 2.9 (0.5, 9.2)	4.7 (12.2); 0.0 (0.0, 2.1)
No movements^c^	1.9 (2.2); 1.3 (0.0, 3.1)	N/A

*Median and 1st and 3rd quartile are additionally reported if the normal distribution is violated

^a^Not including those 12-year-olds (n = 28) and 18-year-olds (n = 13) who did not brush their inner surfaces at all

^b^Only n = 4 12-year-olds showed circular movements at inner surfaces (min: 16%, max: 74.3%); n = 28 of the 18-year-olds showed circular movements at inner surfaces (min: 1.5%, max = 93.8%)

^c^N = 55 12-year-olds showed tooth contact without any movements (min = 0.2%, max = 99.8%) at inner surfaces. At outer surfaces tooth contact without any movements was observed within n = 129 12-year-olds (min = 0.2%, max = 12.3%). No such data are available for the 18-year-olds

^d^N = 140 12-year-olds (min:0.3%, max:98.2%) and n = 36 18-year-olds (min:0.1%, max:76.8%) showed vertical movements on outer surfaces

6 and Fig. 4.

## Abbreviations

ANOVA: Analysis of Variance
dmf-t/DMF-T Index: Index of decayed missing filled teeth
ICC: Intraclass correlation
MS: Assistant MS
PH: Assistant PH
WP: Assistant WP
KB: Assistant KB
TS: Assistant TS

## Footnotes

1 Hager & Werken GmbH & Co. KG, Duisburg, Germany.

2 GABA GmbH, Lörrach, Germany.

3 TePe D-A-CH GmbH, Hamburg, Germany.
